# Supplementary material for: Pure non-local machine-learned density functional theory for electron correlation
Source: Nat Commun. 2021 Jan 12;12:344. doi: 10.1038/s41467-020-20471-y (PMC7804195; doi:10.1038/s41467-020-20471-y)
Supplement: Supplementary file 1 — Supplementary Information [file 41467_2020_20471_MOESM1_ESM.pdf]

Supplementary Information to

Pure non-local machine-learned density functional theory for electron correlation

Johannes T. Margraf[1,2,a] and Karsten Reuter[1,2]

[1] *Chair for Theoretical Chemistry and Catalysis Research Center, Technische Universität München, Lichtenbergstraße 4, D-85747 Garching, Germany*

[2] *Fritz-Haber-Institut der Max-Planck-Gesellschaft, Faradayweg 4-6, D-14195 Berlin, Germany*

(Dated: 27 November 2020)

[a] Electronic mail: johannes.margraf@ch.tum.de

SUPPLEMENTARY NOTE 1: DENSITY FITTING

The DF coefficients C_Q^A can be computed from standard two-electron integrals (involving the orbital and DF basis sets) and the density matrix elements $D_{\mu\nu}$.[1] In the following we will drop the atom index A for convenience. Using the two-electron integrals:

$$(\mu\nu|P) = \iint \chi_\mu(r_1)\chi_\nu(r_1)\frac{1}{r_{12}}\phi_P(r_2)dr_1dr_2 \tag{1}$$

and

$$(P|Q)^{-1} = \left(\iint \phi_P(r_1)\frac{1}{r_{12}}\phi_Q(r_2)dr_1dr_2\right)^{-1}, \tag{2}$$

the DF coefficients are computed as:

$$C_Q = \sum_{\mu,\nu} d_{\mu\nu}^Q D_{\mu\nu}, \tag{3}$$

with

$$d_{\mu\nu}^Q = \sum_P (\mu\nu|P)(P|Q)^{-1} \tag{4}$$

.

SUPPLEMENTARY NOTE 2: ROTATIONALLY INVARIANT DENSITY REPRESENTATION

Applying an arbitrary rotation $\hat{R}$ to a density expanded in a DF basis will change the coefficients C_Q^A, so that:

$$\hat{R}\mathbf{C} = \mathbf{C}', \tag{5}$$

where $\mathbf{C}$ is a vector containing the coefficients.

The DF basis-sets used herein consist of radial and angular components defined as Gaussian Type Orbitals (GTOs) and spherical harmonics, respectively. All basis functions that consist of the same radial function and have the same order spherical harmonics form a so-called shell. Upon application of $\hat{R}$, the coefficients within a shell can be recomputed

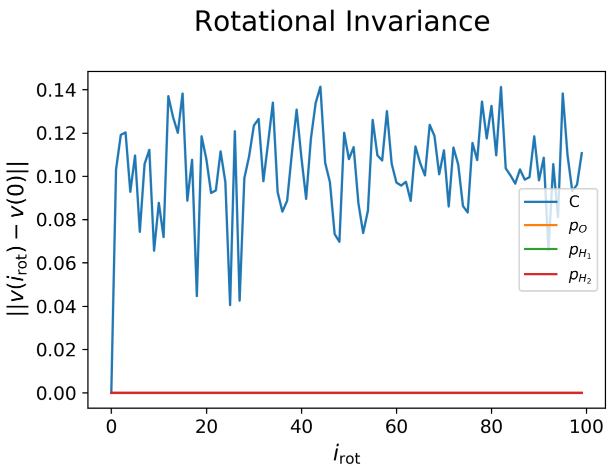

Supplementary Figure 1. Differences of DF coefficients (C) and power spectrum vectors (p) for a randomly rotated water molecule. The differences for the three power spectrum vectors for O and the two H atoms are identically zero.

as linear combinations of the original coefficients in that shell. To obtain a rotationally invariant representation, we can sum the squares of all coefficients belonging to a shell:

$$p_{A,s} = \sum_{Q \in s} (C_Q^A)^2 \tag{6}$$

The set of invariants $p_{A,s}$ for atom A forms the power spectrum vector p_A.[2] With this, we have a highly compact, rotationally invariant representation of the atomic electron density contribution ρ_A.

SUPPLEMENTARY NOTE 3: KERNEL RIDGE REGRESSION

Given the density kernel $K(\rho_i, \rho_j)$ and a training set of N structures with known correlation energies E_c^{ref}, we can define the $N \times N$ kernel matrix $\mathbf{K}$ with the elements $K_{ij} = K(\rho_i, \rho_j)$. We want to obtain the set of coefficients α (see eq. 3) that minimize the regularized least-squares problem:

$$L = \sum_{j}^{N} (E_c[\rho_j] - E_{c,i}^{\mathrm{ref}})^2 + \sigma \alpha^T \mathbf{K} \alpha \tag{7}$$

Defining the vector $\mathbf{y}$, which contains all reference energies E_c^{ref}, the optimal coefficients can be obtained :

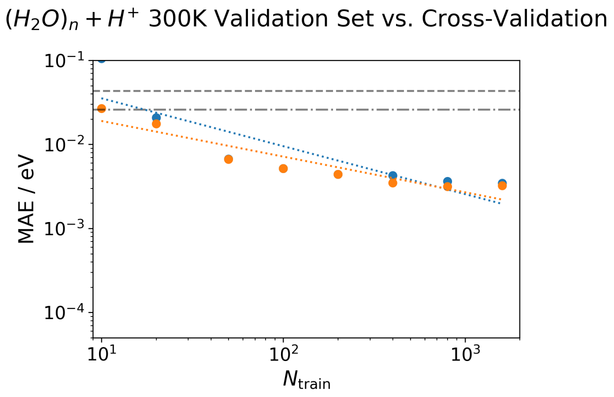

Supplementary Figure 2. Learning curves for the protonated water dimer using ten-fold cross-validation (blue) and a separate validation set (orange) to optimize the regularization hyperparameter.

$$\alpha = (\mathbf{K} + \sigma \mathbf{1})^{-1}\mathbf{y} \tag{8}$$

The optimal regularization parameter σ is determined by optimizing the mean absolute error in predictions on a validation set of 100 structures. Alternatively, cross-validation can be used, though this leads to slightly inferior performance on the smallest (N =10) training sets (see Fig. 2).

As noted previously, KRR models can be used to learn different quantum mechanical reference methods with similar accuracy. For the MP2 and CCSD(T) methods used in the main manuscript, this is shown in Fig. 3.

SUPPLEMENTARY NOTE 4: MONTE CARLO RESAMPLING

The Monte Carlo Resampling (RSM) method allows resampling an ensemble of configurations (e.g. from an MD trajectory) generated at a given level of theory to obtain an approximation to the ensemble at a higher level of theory. To this end, samples are drawn from the original ensemble with replacement. A newly drawn sample c_2 is compared to the previously drawn one c_1 with a Metropolis-like acceptance test:

$$P^{\text{resamp}}_{\text{accept}}(c_1 \rightarrow c_2) = \min(1, \exp[-\frac{\Delta\Delta E}{k_B T}])$$

with

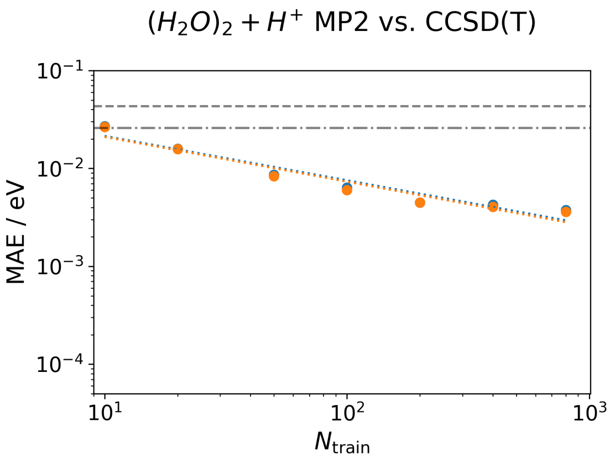

Supplementary Figure 3. Learning curves for the protonated water dimer using MP2 (blue) and CCSD(T) (orange) reference data.

$$\Delta\Delta E = (E_{\mathrm{HL}}(c_2) - E_{\mathrm{LL}}(c_2)) - (E_{\mathrm{HL}}(c_1) - E_{\mathrm{LL}}(c_1)),$$

where HL and LL indicate the energies of the high and low levels of theory.

According to the acceptance test, either c_1 or c_2 is added to the HL ensemble and a new sample is draw. The free-energy surface in Fig. 4 is obtained from a resampled ensemble of 5 million configurations.

SUPPLEMENTARY NOTE 5: COMPARISON WITH GEOMETRY-BASED ML

The overwhelming majority of ML applications in chemistry use a representation of the molecular geometry as input.[3] Compared to the proposed density functional approach, this has the advantage that no electronic structure (e.g. Hartree-Fock) calculations have to be performed at prediction time to obtain the electron density. On the other hand, the KDFA method takes advantage of the fact that the Hartee-Fock energy is automatically computed in addition to the density, so that only the correlation energy has to be learned from data. Overall, one would therefore expect a trade-off: to reach the same accuracy, a KDFA will need fewer training examples than a geometry-based ML model, but it will be more expensive to evaluate at prediction time. If the reference data is calculated with a high-level reference method (e.g. coupled cluster theory) and the target systems are small

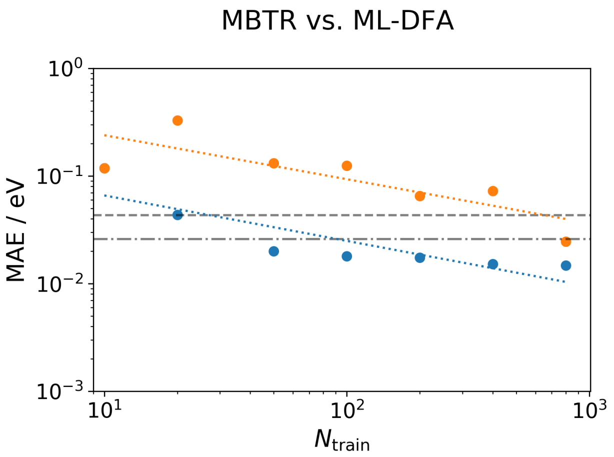

Supplementary Figure 4. Comparison of a geometry based MBTR model (orange) and a KDFA (blue) for the water tetramer.

enough that a mean-field electronic structure calculation (e.g. at the HF level) is affordable, this trade-off favors the KDFA. In contrast, a model trained on DFT data and applied to very large systems would clearly be more efficient using a geometric representation.

There are a wide range of geometric representations and ML models, a comprehensive assessment of which is beyond the scope of the current work. However, to provide quantitative support for the above discussion, we fitted a KRR model for the water tetramer using the Many-Body-Tensor Representation (MBTR) of Huo and Rupp as a representative example of state-of-the-art geometry-based ML.[4] As can be seen in Fig. 4, the MAE for the geometry based model is on average an order of magnitude larger than for the KDFA model. From a different perspective, to reach a similar accuracy as the KDFA, one requires an order of magnitude more training examples.

SUPPLEMENTARY REFERENCES

[1]A. Grisafi, A. Fabrizio, B. Meyer, D. M. Wilkins, C. Corminboeuf, and M. Ceriotti, ACS Cent. Sci. **5**, 57 (2019).

[2]A. P. Bartók, R. Kondor, and G. Csányi, Phys. Rev. B **87**, 184115 (2013).

[3]O. A. von Lilienfeld, Angew. Chemie Int. Ed. **57**, 4164 (2018).

[4]H. Huo and M. Rupp, (2017), arXiv:1704.06439.
