# Supplementary material for: Pure non-local machine-learned density functional theory for electron correlation
Source: Nat Commun. 2021 Jan 12;12:344. doi: 10.1038/s41467-020-20471-y (PMC7804195; doi:10.1038/s41467-020-20471-y)
Supplement: Supplementary file 3 — Description of Additional Supplementary Files [file 41467_2020_20471_MOESM3_ESM.pdf]

Description of Additional Supplementary Files

File Name: Supplementary Data 1
Description: Geometries and reference energies for all models shown in the manuscript.
